# Ipsilateral irradiation for well lateralized carcinomas of the oral cavity and oropharynx: results on tumor control and xerostomia

**DOI:** 10.1186/1748-717X-4-33

**Published:** 2009-09-01

**Authors:** Laura Cerezo, Margarita Martín, Mario López, Alicia Marín, Alberto Gómez

**Affiliations:** 1 Department of Radiation Oncology, Hospital Universitario de la Princesa, Universidad Autónoma de Madrid, Madrid, Spain; 2 Department of Medical Physics, Hospital Universitario de la Princesa, Universidad Autónoma de Madrid, Madrid, Spain

## Abstract

**Background:**

In head and neck cancer, bilateral neck irradiation is the standard approach for many tumor locations and stages. Increasing knowledge on the pattern of nodal invasion leads to more precise targeting and normal tissue sparing. The aim of the present study was to evaluate the morbidity and tumor control for patients with well lateralized squamous cell carcinomas of the oral cavity and oropharynx treated with ipsilateral radiotherapy.

**Methods:**

Twenty consecutive patients with lateralized carcinomas of the oral cavity and oropharynx were treated with a prospective management approach using ipsilateral irradiation between 2000 and 2007. This included 8 radical oropharyngeal and 12 postoperative oral cavity carcinomas, with Stage T1-T2, N0-N2b disease. The actuarial freedom from contralateral nodal recurrence was determined. Late xerostomia was evaluated using the European Organization for Research and Treatment of Cancer QLQ-H&N35 questionnaire and the National Cancer Institute Common Terminology Criteria for Adverse Events (CTCAE), version 3.

**Results:**

At a median follow-up of 58 months, five-year overall survival and loco-regional control rates were 82.5% and 100%, respectively. No local or contralateral nodal recurrences were observed. Mean dose to the contralateral parotid gland was 4.72 Gy and to the contralateral submandibular gland was 15.30 Gy. Mean score for dry mouth was 28.1 on the 0-100 QLQ-H&N35 scale. According to CTCAE v3 scale, 87.5% of patients had grade 0-1 and 12.5% grade 2 subjective xerostomia. The unstimulated salivary flow was > 0.2 ml/min in 81.2% of patients and 0.1-0.2 ml/min in 19%. None of the patients showed grade 3 xerostomia.

**Conclusion:**

In selected patients with early and moderate stages, well lateralized oral and oropharyngeal carcinomas, ipsilateral irradiation treatment of the primary site and ipsilateral neck spares salivary gland function without compromising loco-regional control.

## Background

Radiation therapy is an effective treatment for head and neck cancer patients, showing a high success rate in the early stages of disease. However, permanent xerostomia is a common complication, frequently compromising nutrition and speech, and accelerating dental decay [1]. Xerostomia is caused from bilateral irradiation of the major serous-producing glands, mainly the parotids, and the minor salivary glands which significantly contribute to mucinous secretion [2]. There is not yet an effective treatment for this late complication once it has occurred, thereby, reducing the patient's quality of life.

Head and neck squamous cell carcinomas (HNSCC) are characterized by a relatively orderly spread to regional cervical lymph nodes. Generally, elective neck irradiation is not recommended when the risk of subclinical disease is less than 15-20% [3]. There is growing evidence in the literature that patients with early oropharyngeal and oral cavity cancer have a low incidence of contralateral node involvement, hence, radiation therapy can be limited to the ipsilateral neck, without compromising loco-regional control [4-7]. However, bilateral neck irradiation continues to be the standard approach for most patients, especially those with ipsilateral clinical node-positive presentation. One argument for the continued inclusion of the contralateral neck is that morbidity is low using Intensity Modulated Radiation Therapy (IMRT), because parotid sparing can be easily achieved with this technique [8,9]. However, IMRT is not yet universally available, and, certainly, morbidity will be still lower if only one side of the neck is treated.

In our department three-dimensional conformal radiation therapy (3D-CRT) was started a decade ago, and guidelines for unilateral elective nodal irradiation in patients with HNSCC were implemented soon after [10].

The purpose of the current study was to report on morbidity and tumor control for patients with well lateralized squamous cell carcinomas of the oral cavity and oropharynx treated with the ipsilateral technique. These results will contribute to some previous experiences supporting this conservative approach.

## Methods

### Patients

Twenty patients with early stage HNSCC, where the risk of contralateral neck node involvement was estimated to be less than 15-20%, [3,11-13] were treated with unilateral irradiation between 2000 and 2007. The guidelines for inclusion in the unilateral protocol were as follows: histologically confirmed squamous cell carcinoma; location of the lesion in the tonsillar region with less than 1 cm of medial extension to the soft palate or to the base of the tongue, retromolar trigone, lateral alveolar ridge, cheek mucosa or lateral border of tongue; tumor stage T1-T2 and nodal stage N0, N1, N2a or N2b, according to TNM classification of the UICC-AJCC [14]. Patients with N2 disease up to two ipsilateral nodes, less than 2 cm in diameter, were included in the study, but not those with three or more nodes.

Patients were assessed by clinical examination, by both the head and neck surgeon and the radiation oncologist, endoscopy and CT scan of the head and neck region. A chest X-ray or chest CT and blood test were performed to rule out distant metastases. Patients were treated with primary or postoperative RT with curative intent. Postoperative RT was given to patients with oral carcinomas presenting close (less than 5 mm) or positive margins, or for cases of extracapsular nodal extension in the pathological specimen. Two patients received postoperative chemotherapy, concomitantly with RT. Table 1 shows the demographic, tumor and treatment characteristics of patients. No patient in the present series was treated with contralateral neck dissection. The study was approved by the ethical committee of the hospital, and informed consent was obtained from all patients.

**Table 1:** Demographic, tumor and treatment characteristics of the 20 patients

Characteristics	Number (%)
Age, mean (range) 60 (31- 94)	20 (100%)
Sex	12 (60%)
M	12 (60%)
F	8 (40%)
Tumor site	
**Oral cavity**	**12 (60%)**
Lateral border of tongue	6 (30%)
Retromolar trigone	2 (10%)
Lateral alveolar ridge	3 (15%)
Cheek mucosa	1 (5%)
**Oropharynx**	**8 (40%)**
Tonsil	5 (25%)
Tonsillar pillar	3 (15%)
T stage	
T1	6 (30%)
T2	12 (60%)
T4*	2 (10%)
N stage	
N0	11 (55%)
N1	4 (20%)
N2a-b	5 (25%)
Radiation treatment	
Primary	8 (40%)
Postoperative	12 (60%)
Concomitant chemotherapy	
Yes	2 (10%)
No	18 (90%)

* Two patients with alveolar ridge and retromolar trigone carcinomas with bone erosion.

### Radiotherapy technique

All patients were treated using 3D-CRT. The high dose volume included the gross tumor or the surgical tumor bed with 5 mm set-up margin (PTV1). The elective target included the elective ipsilateral nodal levels with 5 mm set-up margin (PTV2). The guidelines used for the selection of ipsilateral nodal target volume are described in Table 2. The total dose prescribed to the primary tumor was 66-70 Gy for patients with gross disease and 54-64 Gy for patients treated in the adjuvant setting. For elective radiotherapy, 50 Gy was administered to the ipsilateral regions at risk of subclinical disease (PTV2), both for radical and postoperative radiotherapy. All patients were treated with continuous, conventional fractionation of 2 Gy, one fraction per day, five fractions per week.

**Table 2:** Ipsilateral nodal target volumes

Tumor site	Stage	Nodal levels included
Tonsillar fossa	T1-2 N0 T1-2 N1-2	II, III II-IV, RP*
Lateral border of tongue	T1-2 N0 T1-2 N1-2	Ib, II, III Ia, Ib, II, III, IV
Retromolar trigone, lateral alveolar ridge, cheek mucosa	T1-2 N0 T1-2 N1-2	Ib, II, III Ia, Ib, II, III,IV

RP: retropharyngeal lymph nodes

**Figure 1:**
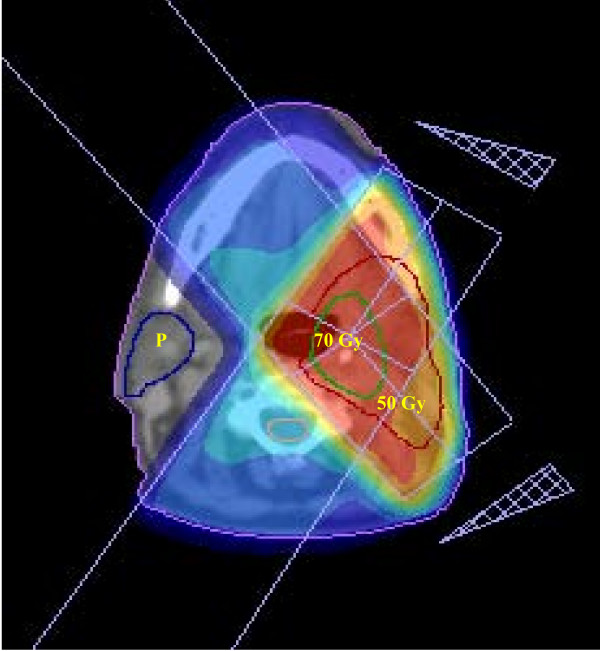
**Oropharyngeal cancer**. A representative example of a CT-based dose plan for a patient with a T2N0M0 tonsillar carcinoma treated with a pair of ipsilateral wedged fields. Green line: PTV1, treated to 70 Gy; red line: PTV2 including ipsilateral II, III and retropharyngeal lymph node levels, treated with 50 Gy. Contralateral parotid and part of the oral cavity are preserved from significant radiation.

**Figure 2:**
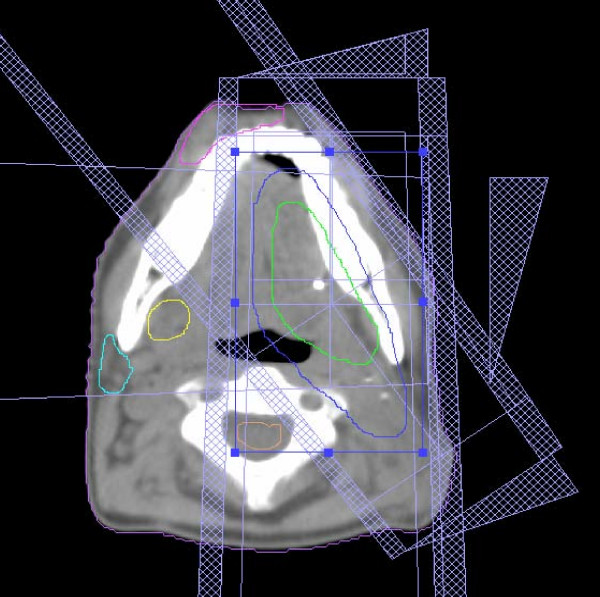
**Oral cavity cancer**. Patient with a pT2N0M0 carcinoma of the left lateral border of tongue treated with postoperative radiation therapy for close margin and perivascular- perineural invasion. Ipsilateral technique using three ports: anterior, left posterior oblique and left lateral. Green line: PTV1, treated to 60 Gy; blue line: PTV2 including ipsilateral Ib, II and III node levels, treated to 50 Gy; cyan line: contralateral parotid, yellow line: contralateral submandibular; mean dose to the right parotid 8 Gy, mean dose to the submandibular gland 20 Gy.

The contralateral parotid and submandibular glands, as well as the spinal cord, were outlined on the planning CT-scan. The goal of treatment planning was maximal exclusion of the contralateral parotid gland, while providing adequate coverage of the target. The most common arrangement used was a two- or three-field ipsilateral technique (Figures 1 and 2). In some cases, where the PTV was more medial, a contralateral field was used to increase the dose homogeneity of the deep part of the target, always sparing the parotid gland. The use of wedges in some fields was common.

### Follow-up

Patients were followed every 3-4 months during the first two years, every 6 months until 5 years, and once yearly until 10 years. Radiation oncologists and head and neck surgeons performed a clinical examination. A head and neck CT or MRI, examination under general anesthesia and/or biopsy were performed if recurrence was suspected.

### Assessment of xerostomia and quality of life

All patients were contacted by phone and given an appointment to assess their morbidity. They completed the EORTC QLQ-H&N35 questionnaire [15] where items are rated on a four-point scale and normalized to a number between 0 and 100. Higher scores represent worse symptoms. The questionnaire was translated for use among Spanish patients [16]. Items 41 (Have you had a dry mouth?) and 42 (Have you had sticky saliva?), directly related to xerostomia, and item 37 (Have you had problems swallowing solid food?), related to dysphagia, and indirectly related to xerostomia, were analyzed in the present study.

Xerostomia was also graded according to the Common Terminology Criteria for Adverse Events (CTCAE) radiation morbidity grading scale, version 3.0 [17]. The CTC evaluation of xerostomia included subjective patient rating, and objective measurement of the unstimulated salivary output, collecting the saliva spit by the patient into a plastic cup for five minutes at least two hours after breakfast. The saliva was weighed on a precision balance and then saliva flow was calculated assuming 1 g saliva was equal to 1 ml saliva [18].

No salivary stimulating or protective agents such as pilocarpine or amifostine were allowed during the study.

### Statistical analysis

Survival data and loco-regional control rates were analyzed from the initiation of radiation treatment using the Kaplan-Meier method. A descriptive analysis was used for the toxicity data. SPSS 16.0 for Windows was used for the statistical analysis.

## Results

### Patients

Twenty patients consecutively treated with unilateral radiotherapy in 2000-2007 were included in this study. No patients treated in this period with the unilateral technique were excluded from the analysis. Eight patients (40%) underwent primary RT while 12 patients (60%) underwent postoperative RT. All patients had T1-T2 squamous cell carcinomas, except two patients with alveolar ridge and retromolar trigone carcinomas, respectively, that were staged pT4 because minimal bone invasion was found in the surgical specimen. Eleven patients were node negative and 9 patients had N1 or N2 disease.

### Dose distribution

According to dose-volume histograms, the mean dose administered to the contralateral parotid gland was 4.72 Gy (range, 1-10 Gy) and to the contralateral submandibular gland 15.30 Gy (range, 1-37 Gy). At least 95% of the target volumes received 97%-105% of the prescribed dose. Mean dose to the PTV1 was 67.5 Gy (range, 64-71 Gy) for primary RT and 58 Gy (range, 54-64 Gy) for postoperative RT. The mean dose to the PTV2 (elective ipsilateral lymph nodes) was 51 Gy (range, 49-52 Gy). The average mean dose to the spinal cord was 8 Gy (range, 1-18 Gy).

### Disease control

With a median follow-up of 58 months, the 5-year overall survival and loco-regional control rates were 82.5% and 100%, respectively. No loco-regional recurrences were found in these patients. Six patients developed a second primary cancer at a median follow-up of 3 years (range, 2-6 years), four of whom died. Pulmonary non-small cell carcinoma was the most frequent type (3 cases), followed by hepatocellular carcinoma (2 cases) and anal carcinoma (1 case).

### Xerostomia

Sixteen patients (80%) filled out the EORTC QLQ H&N35 questionnaire at the concerted visit, at least 1 year after the completion of RT. Mean score for dry mouth was 28.1 on a scale of 0-100, and 26.5 for sticky saliva; mean score for dysphagia was 4.6 on the same scale (Table 3).

**Table 3:** Xerostomia scores from the EORTC QLQ H&N35 scale

Scale item	Mean	Median	Range
Dry mouth (item 41)	28.1	25	(0-50)
Sticky saliva (item 42)	26.5	25	(0-50)
Dysphagia (item 37)	4.6	0	(0-25)

Results from 16 patients who were alive at last follow-up. The QLQ scores were normalized to a number between 0 and 100. Higher numbers, worse symptoms.

When evaluating patients according to the CTCAE v3.0 classification at the same visit, 5 patients (31.2%) had grade 1 xerostomia and 9 patients (56.2%) had no xerostomia symptoms. Two patients (12.5%) showed grade 2 xerostomia. No grade 3 subjective xerostomia was found among these patients (Table 4).

**Table 4:** Frequency and grade of xerostomia according to CTCAE v3.0 scale

Endpoint	Grade 0 N (%)	Grade 1 N (%)	Grade 2 N (%)	Grade 3 N (%)
*Subjective*				
Xerostomia	No complains of xerostomia	Dry or thick saliva	Significant dietary alteration	Inability to adequately aliment orally
	9 (56.2%)	5 (31.2%)	2 (12.5%)	0 (0%)
				
*Objective*				
Salivary flow	> 0.2 ml/min	> 0.2 ml/min	0.1-0.2 ml/min	< 0.1 ml/min
	*	13 (81.2%)	3 (18.7%)	0 (0%)

Results from 16 patients who were alive at the evaluation date;

* Unstimulated salivary flow for Grade 1 is equal to Grade 0

**Figure 3:**
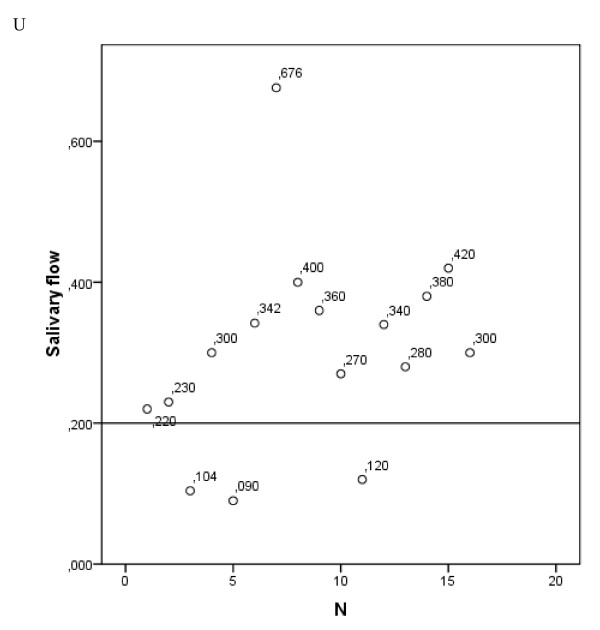
**Salivary flow rates**. Unstimulated salivary flow rates in ml/min in 16 available subjects at least 1 year after treatment. Above the horizontal bar are 13 patients with normal salivary flow ≥ 0.2 ml/min. Only three patients are located below the horizontal bar, with salivary flow < 0.2 ml/min (grade 2 toxicity).

Unstimulated saliva flow was > 0.2 ml/min (grade 0-1 xerostomia) in 13 patients (81.2%), and 0.1-0.2 ml/min (grade 2 xerostomia) in 3 patients (18.7%). No grade 3 objective xerostomia (< 0.1 ml/min) was found in the measurements (Table 4, Figure 3).

## Discussion

Morbidity resulting from irradiation in the head and neck area can be reduced significantly by a comprehensive definition of the CTV, i.e. excluding the contralateral neck in a selected group of patients. A double objective was intended with the ipsilateral technique applied in the present study: to preserve salivary gland function while maintaining loco-regional control. The first was achieved given no grade 3 xerostomia and only 12.5% grade 2 subjective xerostomia were found in our series. The absence of loco-regional recurrences, more specifically, the absence of isolated contralateral neck recurrences, demonstrated the second.

The major benefit of ipsilateral radiation treatment is to provide the opportunity for salivary protection by exclusion of the contralateral major salivary glands and part of the oral cavity mucosa. The mean dose of 4.72 Gy administered to the opposite parotid in our study is well below the dose of 26 Gy recommended by most authors to preserve salivary function. Accordingly, the subjective and objective scores for xerostomia reported in our series were low. Eisbruch et al [1] observed a recovery from xerostomia in unilaterally irradiated patients which was accompanied with a compensatory overproduction of saliva in the contralateral parotid and submandibular gland at 12-24 months. Furthermore, Jellema [19] found that the mean dose given to the contralateral parotid gland was the most important prognostic factor for patient-rated xerostomia.

In general, the CTCAE v3.0 proved to be a practical and adequate tool to measure late xerostomia, since it has two components, a subjective one, based on patient complaints, and an objective part, based on unstimulated salivary flow measurements. However, the salivary flow values do not always correspond to the level of symptoms reported by patients. For example, one patient with salivary flow 0.1-0.2 ml/min, corresponding to a grade 2 objective finding, rated her symptoms as only a grade 1 subjective xerostomia and gave a score of 25 for dry mouth on the H&N35 scale. This finding was further analyzed by Jensen et al [20] who found little correlation between patient-assessed symptom scores according to EORTC questionnaires C30 and H&N35 and objective findings, including saliva flow measurements. Eisbruch et al [1] also described a low correlation between symptoms and salivary measurements. They concluded that both subjective side effects questionnaires and measurement of the saliva should be included in the xerostomia evaluation. As the main objective of minimizing side effects is to improve patient quality of life, subjective symptoms are more relevant, at least in clinical practice.

As expected, this group of patients fared well in terms of loco-regional control and survival, since their tumor burden was low. Generally, elective neck irradiation is not recommended where the risk of subclinical disease is less than 20%, because of the morbidity of radiotherapy. The results of the present study demonstrate that the failure rate in the opposite neck is rare in selected cases with well lateralized tumors of the tonsillar region and oral cavity. In fact, no contralateral neck recurrences were found in any of the twenty treated patients. Jackson [4] and O'Sullivan [5] found similar results, with 2.2% and 3.5% contralateral failure rates, respectively, in two large oropharyngeal cancer series that also included some N+ patients. Other authors reporting on oral cavity and oropharyngeal cancer found a low incidence of contralateral nodal failure (0-3%), although studying fewer patients [6,7,21].

There are frequent scenarios where unilateral irradiation can be applied when treating HNSCC. In the oral cavity, surgery is the most frequent treatment for T1-T2 tumors, however, if margins are positive or close, or if invaded lymph nodes are found, postoperative radiotherapy is commonly indicated to reduce the risk of loco-regional recurrence. Although some authors [8,9] recommend bilateral irradiation when nodal invasion is found in the ipsilateral neck, perhaps a watching policy with close follow-up can be adopted, since the risk for contralateral metastases is still low. The recent report by Rusthoven et al on 20 patients with node-positive tonsil cancer treated with ipsilateral technique and without contralateral neck recurrence illustrates this approach well [22].

Some radiation oncologists are still reluctant to spare the contralateral neck in head and neck cancer patients. This prejudice can be originated in the former standard techniques, because a pair of parallel lateral fields assured good coverage of the target in the bi-dimensional radiotherapy era. Thus, bilateral elective neck irradiation remains the prevailing option for many tumor sites and stages. However, the better knowledge of the pattern of nodal invasion and the advent of three-dimensional planning has brought along higher precision in the delineation of targets, including the nodal targets in the neck. This may allow a progressively conservative tendency in the head and neck radiation treatment. Furthermore, it is feasible that the indications for unilateral techniques will broaden in the near future, once experience is gained for the various tumor sites.

Two patients treated in the last year of the study received postoperative chemoradiation, based on high risk pathological factors: extracapsular nodal invasion in one patient and positive resection margin in the other. The objective of adding concurrent chemotherapy in these patients was to increase the loco-regional control probability. As the recommendations for postoperative chemoradiation are relatively recent [23], we found only one publication on patients with high associated risk factors treated with ipsilateral irradiation plus chemotherapy [22]. This should be further investigated in future studies.

A key question when considering unilateral irradiation, apart from local tumor extension and nodal status, is how a possible contralateral recurrence will be managed. Advances in radiographic and PET imaging have made staging and subsequent follow-up more accurate, allowing for better detection of occult contralateral lymph node metastases. A neck dissection can usually be performed with little morbidity if an isolated contralateral nodal recurrence occurs. However, patients should be involved in the decision to use this approach when the risk is moderate (e.g. those with established regional nodal disease). A close follow-up program is mandatory in these patients in order to diagnose and rescue a possible recurrence as soon as possible.

The observed incidence of late grade 2 xerostomia in the present series compared favorably with other reports of patients treated with parotid-sparing bilateral IMRT [7,24]. This was likely related to the combined sparing of the contralateral parotid and part of the contralateral submandibular gland. Certainly, the salivary function can be further preserved using ipsilateral IMRT because the major salivary glands and some part of the oral cavity can be avoided by the radiation ports. In this regard, Parvathaneni et al [25] have reported on the superiority of IMRT over the wedge pair technique for unilateral treatment of tonsil carcinoma in terms of parotid sparing and conformality of the dose, although the mean dose to the contralateral submandibular gland was not significantly different. As the 3D-CRT ipsilateral technique is simpler to perform and gives acceptable good results, it seams reasonable to reserve IMRT for more advanced stages for whom bilateral neck irradiation is deemed necessary.

Additional methods reported to improve salivary production and reduce xerostomia include protection of the salivary glands by daily amifostine during RT [26] or stimulation with pilocarpine [27]. These methods could only be complementary to planning efforts aimed at reducing the dose given to the major salivary glands and to the oral cavity. For example, Burlage et al [27] reported some benefit of prophylactic pilocarpine when the parotid gland was irradiated with a mean dose above 40 Gy.

Other functions like swallowing can be better kept with the ipsilateral technique since less healthy tissues, like the pharyngeal constrictor muscles, are irradiated. In our series only 3 patients had some problem swallowing solid food, while the rest had no complaints. Swallowing function is closely related with salivation, and the reduced rate of dysphagia in these patients could have been influenced by the normal salivary function in most of them.

One could also hypothesize that morbidity of the radiation treatment may influence overall survival in head and neck patients, since xerostomia can cause malnutrition, dental infections and other debilitating conditions. Some authors comparing ipsilateral and bilateral irradiation in larger series have found better overall survival within the ipsilateral treatment group [6].

## Conclusion

In summary, using an ipsilateral technique in selected patients with well lateralized squamous cell carcinoma of the oral cavity or oropharynx reports clinical benefits, sparing the salivary gland function without compromising loco-regional control. Although the outcomes with ipsilateral RT in the present series were promising, these findings require validation in a larger patient cohort, especially for oral cavity cancer.

## Competing interests

The authors declare that they have no competing interests.

## Authors' contributions

LC designed the study and drafted the manuscript. MM participated in the design of the study and performed the statistical analysis. ML treated some of the patients included in the study and participated in the critical discussion of the data. AM helped draft the manuscript. AG revised the clinical dosimetries. All authors improved the manuscript and approved the final version.
